# Feasibility of an ACT-based group intervention for Malaysian breast cancer survivors with chronic pain

**DOI:** 10.3389/fpsyg.2025.1721854

**Published:** 2026-01-12

**Authors:** Jia Hang Lim, Mahadir Ahmad

**Affiliations:** 1 Faculty of Psychology and Social Sciences, University of Cyberjaya, Cyberjaya, Malaysia; 2 Centre for Community Health Studies (ReaCH), Faculty of Health Sciences, Universiti Kebangsaan Malaysia, Bangi, Malaysia

**Keywords:** intervention, breast cancer, pain acceptance, clinical health psychology, women’s health

## Abstract

**Introduction:**

Breast cancer survivors frequently experience chronic pain, which can impair daily functioning and overall quality of life. This single-arm feasibility study examined the acceptability and preliminary effects of a group-based Acceptance and Commitment Therapy (ACT) intervention among Malaysian breast cancer survivors with chronic pain.

**Methods:**

Twelve participants were enrolled (age range 45–62 years; *M* = 55.3), and nine completed the full intervention. The programme consisted of eight weekly 90-minute sessions facilitated by a clinical psychologist with a minimum of three years of clinical experience. The intervention targeted key ACT processes, including acceptance of difficult internal experiences, present-moment awareness/mindfulness, and values-guided behavioural action. Acceptability was assessed using qualitative feedback on clarity, relevance, and perceived usefulness.

**Results:**

Participants reported that the intervention was understandable, accessible, and helpful in increasing awareness of the interaction between pain and emotion, as well as in strengthening confidence to manage illness-related distress. Participants also described greater psychological flexibility to live with pain while remaining engaged in personally meaningful activities. Reported barriers included logistical constraints (e.g. travel, scheduling), perceived intensity of weekly sessions, and the need for further cultural adaptation of language and examples. Pain intensity following the intervention was rated at 2.89/10 (SD = 1.27). Pre-intervention pain ratings were not collected; therefore, conclusions regarding change in pain severity over time are limited.

**Discussion:**

Nonetheless, the pattern of feedback and completion suggests that a brief, psychologist-led ACT group is both feasible and acceptable in this population. Overall, these findings support the viability of ACT as a culturally adaptable psychosocial approach for Malaysian breast cancer survivors with chronic pain. Refinements to delivery format and contextual tailoring are recommended, and a randomized controlled trial is warranted to evaluate longitudinal clinical outcomes.

**Clinical trial registration:**

https://www.anzctr.org.au/Trial/Registration/TrialReview.aspx?ACTRN=12624000542594p, identifier ACTRN12624000542594p.

## Introduction

Breast cancer is the most common cancer among women worldwide, accounting for approximately 11.7% of all cancer cases (WHO, 2020). Survival rates have improved because of advances in surgery, chemotherapy, radiotherapy, and hormonal therapy, but many survivors continue to live with chronic pain as a long-term consequence of treatment. Between 25 and 60% of breast cancer survivors report persistent pain even years after treatment completion (Schou Bredal et al., 2014). This pain is not only physical – arising from surgical trauma, radiation fibrosis, and chemotherapy-induced neuropathy – but is also associated with anxiety, depression, fatigue, functional limitation, and poorer quality of life (Coughlin et al., 2021; Doan et al., 2023).

Malaysia reflects this global picture. Breast cancer is the most common cancer among Malaysian women, with an age-standardized incidence rate of 34.1 per 100,000 women (Malaysia National Cancer Registry Report, 2019). Pain is highly prevalent: a Malaysian study reported that approximately 79% of women with breast cancer experienced pain ranging from mild to severe (Zubaidah et al., 2013). At the same time, survivors in Malaysia face additional systemic and cultural barriers to pain management. Access to psychosocial support is uneven, psychological services are not routinely integrated into oncology care, and there is a shortage of trained mental health professionals working in cancer settings (Bergerot et al., 2024; Suhardi et al., 2024). Cultural norms can further normalise suffering, encourage stoicism, and discourage help-seeking by framing psychological distress as a personal weakness (Thong et al., 2024; Yigitbas and Bulut, 2024). These realities mean that many women continue to struggle with distressing, functionally impairing pain despite being “medically treated.”

These challenges highlight the need for pain management approaches that are not only clinically effective but also culturally acceptable, deliverable in local services, and psychologically oriented. Although pharmacological strategies such as opioids, anticonvulsants, and antidepressants are commonly used, 10–20% of patients continue to report inadequately controlled pain (Deng, 2019). Current survivorship and palliative care frameworks, including the National Strategic Plan for Cancer Control Programme (2021–2025) and the National Palliative Care Policy and Strategic Plan (2019–2030), call for integrative, biopsychosocial models of care that address both physical symptoms and psychological suffering (IASP, 2023a, 2023b; Kovačević et al., 2024; Sanft et al., 2024). However, to date, no studies have examined whether such approaches—in particular, Acceptance and Commitment Therapy (ACT)—are feasible or acceptable for Malaysian breast cancer survivors living with chronic pain. This represents a clear and important gap in the literature and in service planning (Ghorbani et al., 2021).

Acceptance and Commitment Therapy (ACT) is a third-wave behavioral therapy developed by Hayes and colleagues that aims to increase psychological flexibility: the ability to remain present and engaged in personally meaningful activities even in the presence of pain, distress, or unwanted internal experiences (Hayes et al., 2006; Hayes et al., 2012). Rather than attempting to eliminate difficult thoughts, emotions, or sensations, ACT helps individuals change how they relate to these experiences. It does so through six interrelated core processes: (i) acceptance of difficult internal experiences rather than avoidance; (ii) cognitive defusion, or stepping back from unhelpful thoughts; (iii) being present, through mindful awareness of the current moment; (iv) self-as-context, or adopting a more flexible sense of self; (v) clarifying personal values; and (vi) committed action in line with those values (Hayes et al., 2012). In chronic pain populations, ACT has been associated with improvements in pain acceptance, functioning, and quality of life, even when absolute pain intensity does not fully resolve (Veehof et al., 2016).

In oncology, ACT has shown benefits for anxiety, depression, distress, and quality of life (Feros et al., 2013; Rost et al., 2012; A-Tjak et al., 2014). Importantly, ACT emphasises values-based living and willingness to experience pain in the service of meaningful roles. This focus on living in line with what matters—for example, caring for family, fulfilling spiritual commitments, or maintaining social belonging—maps closely onto Malaysian collectivist values (Jiang et al., 2024). In this way, ACT is not only theoretically relevant to chronic pain but is also culturally compatible with how many Malaysian women make sense of suffering. Emerging work in pain neuroscience further supports this approach: cognitive-emotional processes, such as fear and catastrophising, modulate pain via central pathways (Melzack and Wall, 1965), and ACT-based processes such as acceptance and reduced struggle with pain may help dampen distress-related amplification of nociceptive signalling (Deldar et al., 2021; Garland et al., 2017).

Taken together, chronic pain is widespread among Malaysian breast cancer survivors, it is under-addressed by current care structures, and it is shaped by cultural beliefs that can both burden and motivate coping. Yet no study has evaluated whether ACT can be delivered in this setting, whether Malaysian patients find it acceptable, or whether it shows any early signal of clinical benefit in this population. This study aims to address that gap. Specifically, this study aims to evaluate the feasibility and acceptability of a group-based ACT program among Malaysian breast cancer survivors with chronic pain, and to explore preliminary signals of change in pain and psychological flexibility.

## Method

### Study design

This study was conducted as a single-arm feasibility study of an Acceptance and Commitment Therapy (ACT) protocol for breast cancer survivors with chronic pain. Consistent with feasibility guidance (Dobkin, 2009; Orsmond and Cohn, 2015), the primary aim was to determine whether the intervention and study procedures were acceptable, deliverable, and suitable for progression to a larger controlled trial. Primary feasibility outcomes included recruitment rate (the proportion of eligible patients who consented to participate), retention (the proportion of enrolled participants who completed the intervention), adherence (session attendance across the eight-session programme), acceptability and satisfaction (participant-reported relevance, clarity, and perceived usefulness of the sessions), and treatment fidelity (the extent to which core ACT content was delivered according to the manual). A priori, retention of ≥70% of enrolled participants through the final session, together with consistent delivery of ACT processes, was considered an acceptable feasibility threshold for future scaling. Recruitment rate was defined as the number of enrolled participants divided by the number of patients approached. Retention rate was defined as the number of participants who completed the final session divided by the number enrolled. Adherence was defined as the proportion of the eight scheduled sessions attended by each participant. Acceptability was assessed through participant-reported usefulness, relevance, and perceived impact of the programme. Fidelity was defined as delivery consistent with the manualised ACT protocol.

Secondary outcomes focused on exploratory indicators of clinical change. Clinical measures such as self-reported pain intensity and psychological processes related to pain coping were collected not to evaluate efficacy, but to provide preliminary signals of change and to inform the choice of outcome measures for a future randomized controlled trial. The intervention comprised eight weekly group-based ACT sessions (Gould et al., 2022; Davoudi et al., 2020). Each session lasted approximately 60–90 min and was delivered by a registered clinical psychologist with a minimum of three years of formal training in ACT in Malaysia, using a manualised protocol to support standardisation and fidelity.

### Sampling and recruitment

A total of 12 breast cancer participants with chronic pain were recruited to join the feasibility study. Recruitment took place between March 2025 until May 2025 in the outpatient oncology clinic. All data, including feasibility indicators and post-intervention feedback, were collected during the same period and at the end of the eight-week intervention. All of the participants were joined as one group for the intervention. According to Lewis et.al (2021), a minimum of 10 participants is suitable to run the feasibility study. Despite ending with 9 participants, the study remains within the acceptable range for feasibility studies. The richness of qualitative feedback and consistency of participant engagement across sessions provided sufficient data to evaluate the acceptability, practicality, and perceived benefit of the intervention. As such, the final sample size, while slightly below the guideline, still allows for meaningful interpretation and fulfills the objectives of a feasibility study.

Participants were recruited from a local university hospital in Klang Valley. This hospital was selected as the recruitment site for this study due to its status as a leading tertiary teaching hospital with a well-established oncology department and breast cancer clinic. This hospital serves a large and diverse patient population from across the Klang Valley, making it a suitable and efficient location for identifying and recruiting participants who meet the inclusion criteria.

Participants were recruited in hospital outpatient oncology settings. Members of the research team approached patients in the waiting area while they were awaiting their medical consultation and introduced the study using a brief explanation and a visual poster. Patients who expressed interest were then screened for eligibility by the researchers, including confirmation of a breast cancer diagnosis, presence of chronic pain, and ability to attend the scheduled group sessions. Individuals who met eligibility criteria were provided with detailed verbal and written study information. Those who agreed to participate provided written informed consent prior to enrolment. The consent rate among eligible and contactable patients was 100%.

The inclusion criteria include: 1. Participants must be able to read and write. 2. Participants who have been diagnosed with breast cancer (stage 2/3) and have completed their primary treatments (e.g., surgery, chemotherapy, or radiotherapy) at least six months prior to participation. 3. Participants who have been experiencing pain for more than three months. 4. Participants must be 18 years old and above. The exclusion criteria include: 1. Individuals who are formally diagnosed with mental disorders or are under psychotropic medication. 2. Patient unable to give consent, i.e., due to mental or disease issues (metastatic spread to the brain, etc.). The decision to include only Stage 2 or 3 survivors was intentional. Patients with Stage 2/3 disease are more likely to undergo multimodal, invasive, curative-intent treatment (e.g., axillary surgery, adjuvant chemotherapy, and radiotherapy), which has been associated with a high burden of persistent neuropathic, musculoskeletal, and post-surgical pain that can continue for months to years after treatment completion and significantly impair functioning and quality of life (Schou Bredal et al., 2014; Zubaidah et al., 2013). This subgroup therefore represents a clinically relevant high-need population. In addition, Stage 2/3 survivors constitute the intended target population for the planned future definitive trial, which aims to evaluate the effectiveness of ACT in women with substantial and enduring pain-related morbidity following breast cancer treatment.

### Intervention adaptation

The intervention was adapted from the “Life with Chronic Pain: An Acceptance-Based Approach” protocol by Vowles and Sorrell (2007) and refined into a structured, group-based Acceptance and Commitment Therapy (ACT) programme. The adaptation process involved expert review by clinicians experienced in psycho-oncology and ACT to ensure cultural relevance for Malaysian breast cancer survivors, with particular attention to language, illness beliefs, family roles, and community reintegration concerns. Content was manualised into an eight-session programme (weekly, 90 min per session; see Table 1), and a corresponding participant workbook was developed to support continuity between sessions.

**Table 1:** ACT intervention and contents.

No. Session	Content
Session 1	Introducing group member and discuss group rules. Provide rationales for the intervention and the approach of the intervention. Homework: Listing down. If pain will be present for the rest of your life, what could you do next?
Session 2	Recap session 1 and discuss assessment of prior session assignment, assessment of patients’ problems from ACT perspective, extraction of avoidance experience, mixing and individual values. Homework: Mood checked-in
Session 3	Recap session 2. Assessment of prior session assignment, discuss acceptance of chronic pain, identifying patients’ life values and evaluating values based on their important level. Homework: Complete the values assessment rating form
Session 4	Recap session 3. Training distinction between evaluations and personal values and action. Teaching mindfulness. Homework: Goals and action worksheet
Session 5	Recap session 4 and practice mindfulness exercises. Highlighting the inefficiency of controlling negative events using metaphors and training the tendency toward negative emotions and experiences Homework: Select one/two actions to perform. Record the results of your performance with regard to values. Practice mindfulness.
Session 6	Recap session 5. Assessment of prior session assignment, planning for action versus action and discuss process of action allow leaning to occur. Homework: Commit yourself to action. Track progress, difficulties, and experiences over the week and take some time daily to review.
Session 7	Recap session 6. Presenting practical solutions to eliminate the obstacles while applying metaphors and planning for commitment to pursue values. Homework: There is no real “homework” this week. You are hopefully at a point of making your own decisions about what works best for you
Session 8	Recap session 7. Summing up the concepts discussed in the previous sessions, asking members to explain their achievements to the group and talking about their future plan.

The intervention incorporated experiential exercises, metaphors, group discussions, and between-session homework. These components were organised to target three core therapeutic aims: (i) increasing awareness and acceptance of thoughts, emotions, and bodily sensations related to cancer and chronic pain; (ii) reducing cognitive rigidity and fusion with distressing or self-limiting beliefs (e.g., “I am no longer useful,” “I am a burden”) by fostering more flexible responding; and (iii) clarifying personally meaningful values and supporting committed action consistent with those values. ACT metaphors and experiential practices were deliberately selected and refined during expert review for their relevance to the psychosocial challenges commonly reported by Malaysian breast cancer survivors, including anxiety about recurrence, fear of social judgement, and difficulty resuming valued roles following treatment.

All sessions were delivered in a private room within the hospital ward. Group size was nine participants. Attendance was high, with all participants attending more than 80% of the scheduled sessions. Each session followed a standardised structure and was supported by printed handouts and a participant activity workbook, which included reflective exercises and values-based action planning. No audio recordings or digital materials were provided for home use. Participants did not receive any concurrent structured psychological intervention during the study period, and no additional individual psychotherapy was delivered as part of, or alongside, the group programme. Standard medical care for cancer follow-up continued as usual, but no adjunct psychological care was introduced by the research team. This reduces the potential confounding influence of other psychosocial inputs on perceived benefit.

The mode of delivery for group Acceptance Commitment Therapy (ACT) involved face-to-face sessions, where a registered clinical psychologist leads a group of participants through various exercises, discussions, and homework assignments. In terms of intervention format, group ACT typically follows a structured protocol. Each session typically includes psychoeducation, experiential exercises, and group discussions. Homework assignments may also be given to help participants practice the skills they have learned in the sessions. The overall goal is to provide a supportive and collaborative environment where participants can learn and practice the skills of ACT together. A total of 8 sessions were conducted. Each session takes approximately 90 min. The clinical psychologist delivered the intervention in accordance with the manual and accompanying worksheets, and participants’ completed worksheets were collected to verify adherence to the protocol.

### Procedure

Before the study commenced, ethical approval was obtained from the Research Ethics Secretariat of university, and all procedures were carried out in accordance with the principles of the Declaration of Helsinki. The study was also registered with the Australian New Zealand Clinical Trials Registry (ANZCTR).

**Figure 1:**
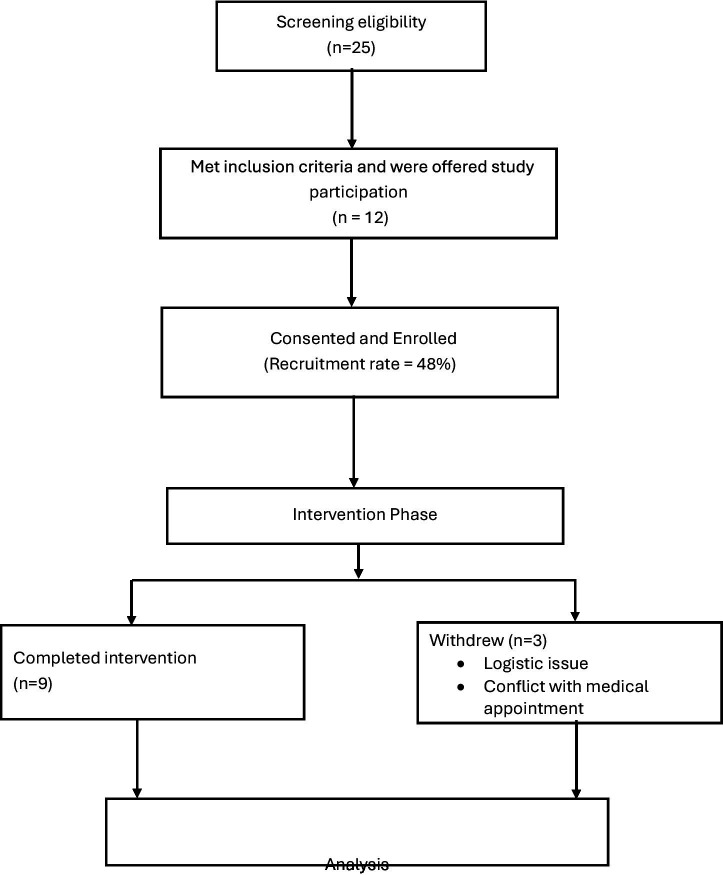
CONSORT-style participant flow diagram.

Participants were thoroughly briefed about the objectives, structure, and expectations of the study in a private and comfortable setting. This session ensured that each participant had ample opportunity to ask questions and clarify any concerns regarding their involvement. After confirming their understanding, participants provided written informed consent, acknowledging their voluntary participation and the right to withdraw at any stage without consequence. Following completion of the 8-session ACT group intervention, participants were requested to complete a feasibility questionnaire (Figure 1). This included structured feedback on the intervention’s relevance, accessibility, and usefulness, as well as the Visual Analog Scale (VAS) to assess changes in pain perception. The questionnaire was administered at the final session to capture immediate reflections and insights gained through participation in the therapy sessions.

### Data analysis

Both quantitative and qualitative analyses were conducted. All outcome data were collected post-intervention only; no baseline (pre-intervention) measures were obtained. This absence of baseline data limits the ability to infer change over time and increases the risk of overestimating perceived improvement, as any post-intervention differences cannot be distinguished from natural recovery, expectancy effects, or social desirability.

Quantitative data were analysed using descriptive statistics (frequencies, percentages, means, and standard deviations). Descriptive summaries were generated for participant demographics, recruitment and retention rates and documented reasons for absence or attrition, as these are core feasibility indicators. Pain intensity was assessed at the end of the programme using a single-item rating (“Place a mark on the line below to indicate your current level of pain”) and was summarised using mean and standard deviation. No inferential statistical tests (e.g., paired t-tests, Wilcoxon signed-rank tests) were conducted because there were no pre–post paired data and the sample size was small; pain ratings are therefore interpreted only as descriptive, exploratory indicators.

Intervention acceptability, relevance, and perceived impact were assessed using a study-specific feasibility questionnaire developed for this project rather than a validated instrument. Items asked participants to comment on: (i) perceived usefulness (“Was the group therapy useful for you? If yes, which parts?”); (ii) relevance (“Was the content of the group therapy relevant?”); (iii) perceived psychological impact (“Did the group therapy make you more confident to fight your disease?”; “Did the group therapy make you more anxious?”); (iv) illness understanding (“Did the group therapy help you better understand your current disease?”); (v) practical facilitators and barriers to attendance (“What is your motivation and what are the barriers to attend such a programme?”); (vi) pain intensity (as noted above); and (vii) open-ended reflections on suggested improvements (“Is there anything that could help us improve the contents of the group therapy?”) and overall experience (“Patient’s experience”). Responses to structured items were summarised descriptively. Narrative responses to open-ended items were analysed qualitatively.

Qualitative data consisted of written free-text responses to open-ended questions and spontaneous participant feedback regarding burden, cultural fit, and perceived usefulness of the sessions. These data were analysed using a reflexive thematic approach. Coding was performed inductively by the research team to identify recurring patterns in perceived benefits, sources of difficulty, and suggestions for optimisation. Triangulation was undertaken by comparing participant accounts with implementer reflections (session notes on engagement, participation, and observed barriers) to strengthen credibility and to identify overlapping feasibility concerns (e.g., scheduling, emotional load) versus isolated individual preferences. Formal inter-rater reliability statistics were not calculated due to the exploratory nature and small sample; instead, credibility was supported through iterative discussion and agreement on final thematic interpretations.

This was a single-arm feasibility study. Quantitative indicators (e.g., recruitment, attendance, retention, pain ratings, and self-reported confidence) were analysed descriptively and were interpreted alongside qualitative themes (e.g., perceived usefulness, cultural relevance, anxiety triggers, logistical barriers) to assess feasibility and acceptability of the intervention and study procedures. These qualitative data were used to contextualise and explain feasibility outcomes, rather than to conduct quantitative–qualitative data integration. Attendance was monitored for each session, and adherence was calculated as the proportion of the eight scheduled sessions attended by each participant. Reasons for missed sessions and attrition were documented to inform future implementation planning. All group sessions were conducted under explicit confidentiality agreements: participants were reminded at the outset not to disclose other members’ personal experiences outside the group. For data handling, questionnaires were de-identified at the point of collection and stored separately from identifying information.

Given the single-arm design, the absence of a comparator group, and the reliance on post-intervention self-report, all quantitative findings should be interpreted cautiously. Any apparent benefits (e.g., increased confidence, lower reported pain, improved understanding) may reflect expectancy, perceived obligation to report improvement, or social desirability in a group setting, rather than a direct effect of the intervention itself.

## Results

### Participants

A total of 25 breast cancer survivors with chronic pain were approached in the outpatient hospital setting. Of these, 12 consented to participate and were enrolled in the study, yielding a recruitment rate of 48%. During the course of the intervention, three participants withdrew due to logistical barriers (i.e., transport and scheduling difficulties) and competing medical appointments, resulting in nine participants completing the full programme. This corresponds to a retention rate of 75% among enrolled participants. The retained sample comprised primarily Malay participants (88.8%), with one Chinese participant (11.1%). The mean age of participants was 55.3 years (range = 45–62 years). Participant demographic characteristics are presented in Table 2.

**Table 2:** Demographic information.

Variable	Category	*n*	%
Age (45–62)	*M* = 55.3		
Ethnicity	Malay	8	88.9
	Chinese	1	11.1
Gender	Female	9	100

Adherence to the intervention was acceptable for a feasibility study. Among participants who completed the programme (n = 9), mean session attendance was approximately 81% of the eight scheduled sessions, corresponding to an average of approximately 6.5 sessions attended per participant. All eight planned sessions were delivered as outlined in the manualised ACT protocol, and session content was delivered by a clinical psychologist with formal ACT training and supervised experience. Fidelity was monitored through adherence to the session manual and review of completed participant worksheets collected at the end of each session, which confirmed that core processes were consistently introduced as intended (Table 3).

**Table 3:** Feasibility metrics for the single-arm ACT feasibility study.

Feasibility metric	*n*	%
Recruitment rate	12	48
Retention rate	9	75
Adherence (Session attendance)	≥5 of 8	*M* = 81

Participants’ self-reported evaluations indicated that the intervention was generally acceptable and perceived as personally meaningful. Most participants (7 of 9) described the group sessions as useful, and just over half (5 of 9) indicated that the material felt relevant to their lived experience of cancer and chronic pain. Four participants reported feeling more confident in managing their condition after the sessions, and five participants stated that the intervention improved their understanding of their current health situation. Five participants also reported feeling motivated to continue attending sessions. These indicators of acceptability were derived from a study-specific, non-validated post-intervention questionnaire and therefore represent subjective perceptions of usefulness, relevance, and perceived impact rather than scores on a standardized satisfaction scale.

Pain intensity was assessed at the end of the programme using a Visual Analog Scale (VAS) ranging from 0 (no pain) to 10 (maximum pain). At post-intervention, participants reported mild to moderate pain levels (M = 2.89, SD = 1.27). Because pain ratings were collected only at the end of the intervention and no pre-intervention baseline was obtained, these values are descriptive and cannot be interpreted as evidence of improvement attributable to the intervention. Any perceived benefit in pain experience or illness-related confidence may reflect non-specific factors such as natural symptom fluctuation, regression to the mean, expectancy/placebo effects, social desirability in self-report, or the supportive nature of a shared group environment rather than specific therapeutic effects of ACT. These data should therefore be regarded as exploratory signals informing future outcome selection, not as efficacy findings.

Qualitative feedback was analysed using a reflexive thematic approach. Free-text responses were inductively coded to identify patterned meanings in participants’ accounts of usefulness, burden, and anticipated barriers to continued engagement. Four dominant themes were identified. First, perceived usefulness: several participants described the sessions as helpful in coping with the emotional and practical challenges associated with chronic pain after cancer. Second, group support: participants valued being in a shared space with other breast cancer survivors, reporting a sense of being understood and less isolated. Third, cultural resonance: participants indicated that the language, metaphors, and values-based discussions aligned with their lived social roles (e.g., family responsibility, duty, spiritual meaning), suggesting cultural fit. Fourth, barriers to participation: barriers clustered into (i) logistical constraints (e.g., travel to hospital, transport dependence), (ii) health-related constraints (e.g., fatigue and physical limitations linked to ongoing cancer-related morbidity), and (iii) medical scheduling demands (e.g., conflicts with routine follow-up appointments). These themes reflect systematically coded patterns and are not presented as anecdotal testimonials.

Feedback from the implementer (i.e., the clinical psychologist delivering the sessions) was documented separately to avoid conflating therapist observation with participant self-report. The implementer noted consistent engagement with acceptance-based and values-based exercises, as well as recurrent expressions of fear regarding cancer recurrence and challenges related to re-integration into family and community roles. The implementer also observed that logistical constraints, ongoing medical fatigue, and clashes with hospital appointments were the most common reasons for missed sessions. These observations were used to contextualise feasibility (e.g., setting, timing, mode of delivery) and to inform potential modifications for future trials.

Overall, these findings provide preliminary evidence that the intervention and study procedures are deliverable in this setting and broadly acceptable to participants. However, they should be interpreted cautiously. The study was a single-arm feasibility design with a small sample size, no comparator group, and no pre–post clinical data. As such, the results cannot be taken as evidence of clinical efficacy. Rather, they are intended to inform procedural optimisation (recruitment, retention, adherence, and fidelity), identify barriers to participation, and guide the design of a subsequent controlled trial.

## Discussion

This single-arm feasibility study provides preliminary evidence on the procedural viability, acceptability, and contextual fit of a group-based Acceptance and Commitment Therapy (ACT) intervention for Malaysian breast cancer survivors living with chronic pain. The findings should be interpreted as exploratory and descriptive, rather than as evidence of clinical effectiveness.

The study demonstrated moderate-to-acceptable feasibility across several domains that are commonly used to judge readiness for a larger trial, including recruitment, retention, adherence, and therapist fidelity. The recruitment rate was 48%, meaning that 12 of 25 approached patients consented to participate. Although this falls slightly below a commonly cited feasibility benchmark of approximately 50% recruitment, it indicates that nearly half of eligible and approached patients were both willing and able to consider a psychosocial intervention embedded within oncology follow-up. Retention was 75%, with nine of 12 enrolled participants completing the programme, which exceeds the commonly applied threshold of ≥70% retention for feasibility progression. Adherence among completers was high: participants attended, on average, approximately 81% of the eight planned sessions, suggesting that the weekly 90-min format was broadly tolerable despite health and logistical constraints. Intervention fidelity was preserved; all eight sessions were delivered as planned by a clinical psychologist, using a manualised protocol, and session worksheets were collected to verify delivery of core ACT processes. Taken together, these indicators suggest that structured, psychologist-led group ACT can be delivered in this setting with acceptable procedural stability, while also identifying areas that require refinement before a definitive trial.

Participant feedback suggested that the intervention was generally perceived as useful and personally relevant. Most participants reported that the sessions helped them reflect on their illness experience and cope with ongoing pain and distress, and several described feeling more confident in managing their condition and better able to understand their current health situation. These subjective reports are broadly consistent with prior evidence that ACT can enhance psychological flexibility, reduce pain interference, and improve emotional functioning in chronic pain populations (Ye et al., 2024), and aligns with work in oncology suggesting that ACT can support adaptive coping, acceptance, and values-based engagement in life despite ongoing symptoms (Song et al., 2024). Participants also described the group format as meaningful, noting that being in a room with other breast cancer survivors reduced feelings of isolation and fostered validation and mutual support. This perceived sense of connection and shared understanding reflects prior findings that peer contact and social normalisation are protective factors in survivorship and adjustment to ongoing symptoms and fear of recurrence (Fox et al., 2023).

Qualitative analysis highlighted three thematic areas with direct relevance to feasibility: (i) empowerment and perceived usefulness, in which participants described gaining a sense of agency in responding to pain and illness-related distress rather than feeling overwhelmed by it; (ii) group connection and shared identity, in which the group context itself was described as emotionally containing and supportive; and (iii) cultural resonance, in which participants indicated that discussions anchored in values, family roles, responsibility, and perseverance felt consistent with their lived realities and social obligations. These themes are consistent with emerging arguments that psychosocial oncology interventions may be strengthened when they explicitly address culturally embedded beliefs about suffering, duty, and survivorship, and when they help patients situate their pain experience within those value systems (Arundell et al., 2021). At the same time, several participants reported difficulty understanding some of the terminology and metaphors in the original ACT materials, indicating that further cultural and linguistic adaptation is necessary to optimise clarity, engagement, and accessibility for Malaysian survivors.

Despite acceptable adherence and retention, participants identified meaningful barriers to consistent participation. These barriers clustered into: (i) logistical burdens, including travel to the hospital, reliance on others for transportation, and clashes with routine medical appointments; (ii) health-related limitations, such as fatigue and physical discomfort linked to ongoing cancer- and treatment-related morbidity; and (iii) scheduling intensity, with some participants noting that weekly sessions felt demanding in the context of their broader recovery and follow-up schedule. These observations mirror recent work showing that logistical constraints, health-related fatigue, and competing clinical demands are common barriers to psychosocial intervention attendance and continuity in cancer populations (Burke et al., 2025). They also align with recommendations for alternative delivery models, including shorter formats, reduced session frequency, and hybrid or remote delivery, which may improve accessibility and retention in future trials (Taylor et al., 2024). Such adaptations are particularly relevant in survivorship care, where symptom burden, travel demands, and caregiving responsibilities may be substantial.

Descriptively, post-intervention pain ratings suggested that most participants reported mild to moderate pain severity at the end of the programme (M = 2.89, SD = 1.27 on a 0–10 scale). These data were collected only at a single post-intervention timepoint and therefore cannot be interpreted as evidence of pain reduction. Nonetheless, they support the clinical relevance of this population: even in survivorship, women continue to report persistent pain that has emotional, functional, and identity implications. This observation is consistent with literature indicating that ACT may contribute to improvements in functional adjustment, pain acceptance, and emotion regulation in individuals with chronic pain (Ye et al., 2024; Wang et al., 2025). However, any apparent improvement in confidence, self-management, or perceived coping in the present study must be interpreted with caution. In the absence of a control group, and without baseline data for comparison, these reports may reflect non-specific therapeutic factors such as expectancy effects, social desirability, perceived obligation to show benefit, or the supportive nature of the group context rather than effects specific to ACT content.

### Limitations

Several limitations should be noted. First, the sample size was small and the study retained nine participants, which limits the precision and generalisability of the findings. Second, this was a single-arm feasibility study with no comparator group and no pre–post assessment of clinical outcomes. As a result, any reports of perceived benefit (e.g., feeling more confident in managing illness, better understanding of one’s condition, or lower distress related to pain) cannot be attributed to the intervention itself. In the absence of baseline data and a control condition, it is not possible to distinguish potential intervention effects from natural improvement over time, regression to the mean, expectancy/placebo effects, or the supportive influence of a shared group setting.

Third, feasibility outcomes were obtained in a single oncology setting and reflect a largely Malay, middle-aged survivorship population, which restricts transferability to younger survivors, non-Malay groups, men, or individuals with different disease stages. Fourth, contextual factors may have influenced both attendance and engagement. Participants frequently reported logistical barriers such as transport burden, clashes with medical appointments, physical fatigue related to ongoing treatment effects, and scheduling demands associated with follow-up care. These real-world constraints likely influenced adherence and may have limited participation among individuals with higher symptom burden or fewer practical supports. More broadly, service delivery conditions (e.g., hospital access policies, clinic scheduling pressures, and broader health system strain in the recovery period following COVID-19) may have affected who was able to attend and remain in the programme.

Moreover, all acceptability and perceived-impact data were self-reported at the end of the programme and are therefore vulnerable to social desirability bias. Lastly, although fidelity was maintained by a single trained clinical psychologist delivering all sessions according to a manualised protocol, this raises the possibility of therapist effects that may not generalise to other facilitators or settings.

### Future directions

The present findings have several implications for the design of a future randomized controlled trial (RCT). First, recruitment and retention data provide realistic estimates for sample size planning, timelines, and resource needs for a definitive trial. In addition, the observed logistical barriers suggest that future work should evaluate adapted delivery models (e.g., reduced session frequency, shortened session length, telehealth or hybrid formats) to enhance reach and reduce attrition (Burke et al., 2025; Taylor et al., 2024). Cultural and linguistic tailoring of intervention materials which including simplifying terminology, embedding locally meaningful metaphors, explicitly addressing fears of recurrence and social reintegration, and acknowledging caregiving obligations appears necessary to ensure comprehensibility and engagement (Arundell et al., 2021). Furthermore, the next-stage trial should incorporate standardised pre- and post-intervention measures of pain interference, psychological flexibility, mood, and role functioning, consistent with prior ACT trials in chronic pain and oncology populations (Ye et al., 2024; Song et al., 2024; Wang et al., 2025). Finally, cost, scalability, and staffing requirements should be examined, including whether delivery can be supported by trained therapists within oncology services or whether alternative delivery models (e.g., co-facilitation, stepped-care approaches) are feasible.

## Conclusion

In summary, this feasibility study suggests that a manualised, group-based ACT intervention was deliverable within an oncology follow-up setting, with acceptable retention (75%), adequate adherence (~81% attendance among completers), and preserved fidelity across all eight sessions. Participants described the programme as useful, relevant, and culturally resonant, and valued the opportunity for connection with peers. At the same time, practical barriers related to transport, scheduling, health status, and language clarity were evident and must be addressed prior to scale-up. These preliminary findings support the acceptability and cultural adaptability of ACT for Malaysian breast cancer survivors with chronic pain. Further controlled trials are needed to determine its clinical efficacy, cost-effectiveness, and optimal mode of delivery in routine survivorship care.

## Data Availability

The raw data supporting the conclusions of this article will be made available by the authors, without undue reservation.
